# In vivo visualization of the i-motif DNA secondary structure in the *Bombyx mori* testis

**DOI:** 10.1186/s13072-020-00334-y

**Published:** 2020-03-05

**Authors:** Wenhuan Tang, Kangkang Niu, Guoxing Yu, Ying Jin, Xian Zhang, Yuling Peng, Shuna Chen, Huimin Deng, Sheng Li, Jian Wang, Qisheng Song, Qili Feng

**Affiliations:** 1 Guangdong Provincial Key Laboratory of Insect Developmental Biology and Applied Technology, Institute of Insect Science and Technology, School of Life Sciences, South China Normal University, Guangzhou, 510631 China; 2 Guangzhou Key Laboratory of Insect Development Regulation and Application Research, Institute of Insect Science and Technology, School of Life Sciences, South China Normal University, Guangzhou, 510631 China; 3 Department of Entomology, University of Maryland, College Park, MD 20742 USA; 4 Division of Plant Sciences, University of Missouri, Columbia, MO 65211 USA

**Keywords:** DNA secondary structure, i-motif, Chromosome, In vivo detection, Epigenetic regulation

## Abstract

**Background:**

A large number of in vitro experiments have confirmed that DNA molecules can form i-motif advanced structure when multiple cytosines exist in the sequence. However, whether these structures are present in vivo environment still lacks sufficient experimental evidence.

**Results:**

In this paper, we report the in vivo visualization of i-motif structures in the nuclei and chromosomes of the testis of the invertebrate *Bombyx mori* using immunofluorescence staining with an antibody specifically recognizing the endogenous transcription factor BmILF, which binds i-motif structure with high specificity. The number of i-motif structures observed in the genome increased when the pH was changed from basic to acidic and decreased under treatment with an i-motif inhibitor, the porphyrin compound TMPyP4. The pH change affected the transcription of genes that contain i-motif sequences. Moreover, there were more i-motif structures observed in the testis cells in interphase than in any other cell cycle stage.

**Conclusions:**

In this study, the i-motif structures in invertebrates were detected for the first time at the cell and organ levels. The formation of the structures depended on cell cycle and pH and affected gene expression.

## Background

DNA is usually present as a double-helix structure with the strict base-pairings of adenine (A) to thymine (T) and cytosine (C) to guanine (G) [1]. However, when multiple Gs or Cs continuously and tandemly exist in double-stranded DNA (dsDNA) or single-stranded DNA (ssDNA) or RNA, the DNA or RNA molecule can form G-quadruplex (G4) or i-motif secondary structures, respectively [2–5]. These G4 and i-motif structures have been found to play important roles in the regulation of many genetic activities including transcription, DNA replication, protein translation and telomere protection [2, 3, 9–13]. The existence and function of secondary structures in DNA and RNA molecules provide a novel epigenetic mechanism for regulating genetic activities [9, 14]. Recent studies in vertebrates and invertebrates suggest that changes in the G4 or i-motif structure (either stabilization/folding or destruction/unfolding) could lead to changes in gene transcription [10, 15, 16].

The i-motif was first discovered by Gehring et al. [17], who found that C-rich DNA can form a quadruple-helical structure by the formation of C–C base pairs via Hoogsteen bonds under acidic conditions. The i-motif is a four-stranded structure formed by intercalated hemiprotonated C–C^+^ base pairs under acidic conditions [4, 6] or at neutral pH [7, 8] by the molecular crowding of the co-solutes or at high pressure. The i-motif structure has been found in the promoter of many genes and can regulate gene transcription in cell lines [6, 9, 10, 14]. The action of the i-motif in the regulation of transcription can be negative [18] or positive [10, 19]. However, more experimental evidence is needed to show the in vivo existence and function of the i-motif secondary structure. While G4 structures have been detected in vivo in several studies [13], the i-motif structure was also observed recently in human cells using state-of-the-art in-cell NMR spectroscopy [20] or using an antibody (iMab) with high selectivity and affinity against the i-motif structure, and the in vivo formation of this structure is cell cycle and pH dependent [16].

In our previous study [10], an i-motif structure was identified in the promoter region of the transcription factor gene *BmPOUM2* in *Bombyx mori*. Another transcription factor BmILF regulates *BmPOUM2* expression by binding to its i-motif structure, as demonstrated by electrophoretic mobility shift assay (EMSA) and DNA chromatin immunoprecipitation (DNA ChIP). In this paper, we report the in vivo visualization of the i-motif structure in the nuclei and chromosomes of the *B. mori* testis by immunofluorescence staining using the BmILF protein and its antibody. The effects of pH, porphyrin compounds and the cell cycle on the formation of the i-motif structure were analyzed.

## Results

### Effect of pH on the formation of the i-motif structure

**Fig. 1:**
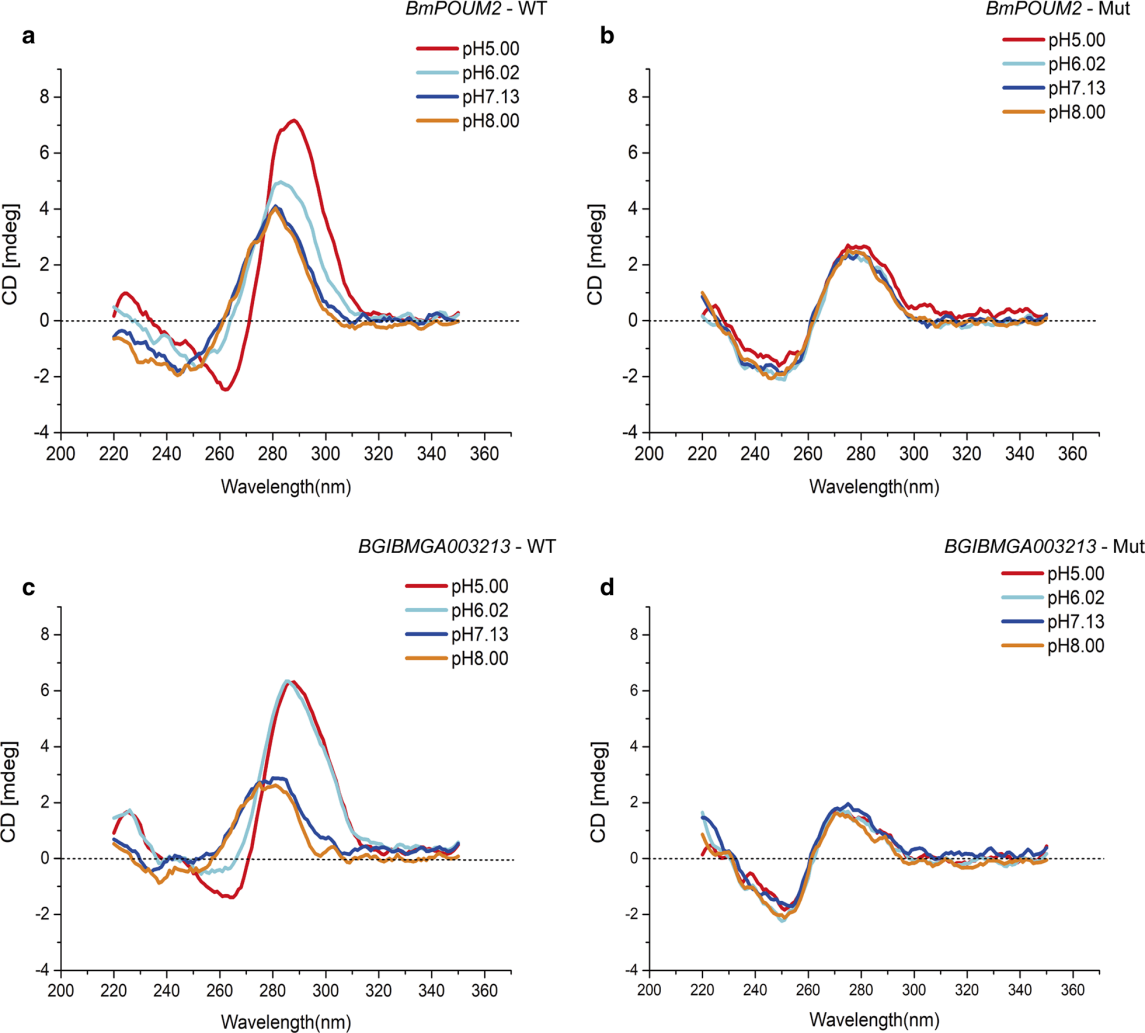
CD analysis of the effect of pH on the formation of i-motif structures. **a***BmPOUM2* wild-type; **b***BmPOUM2* mutant; **c***BGIBMGA003213* wild-type; **d***BGIBMGA003213* mutant. The sequences of these DNA fragments are listed in Table 1. DNA oligonucleotide sequences were folded in Tris–TAE buffer at pH 5.00, 6.02, 7.13 and 8.00 before CD scanning from 200 to 360 nm. The wild-type and mutated sequences are shown in Table 1

To further analyze the effects of pH on the formation of i-motif structures in *BmPOUM2* and an unknown *B. mori* gene (*BGIBMGA003213*) that were predicted based on sequence analysis, wild-type and mutant versions of these sequences were selected for CD analysis at different pH values. The absorption peak of the i-motif structure at 290 nm [6, 9, 14] was detected at pH 5.00, implying that these sequences could form i-motif structures at these pH values (Fig. 1a, c). When the pH value was increased to alkaline conditions, the peaks shifted and decreased in amplitude. When the cytosines in the DNA sequences were mutated, no featured peaks were detected at any pH (Fig. 1b, d), suggesting that the i-motif structures could not form. These results suggested that like the *BmPOUM2*, the *BGIBMGA003213* (hereafter referred to as 3213) gene sequence also contains an i-motif structure whose formation is pH dependent.

### Specific binding of BmILF to i-motif structures

**Fig. 2:**
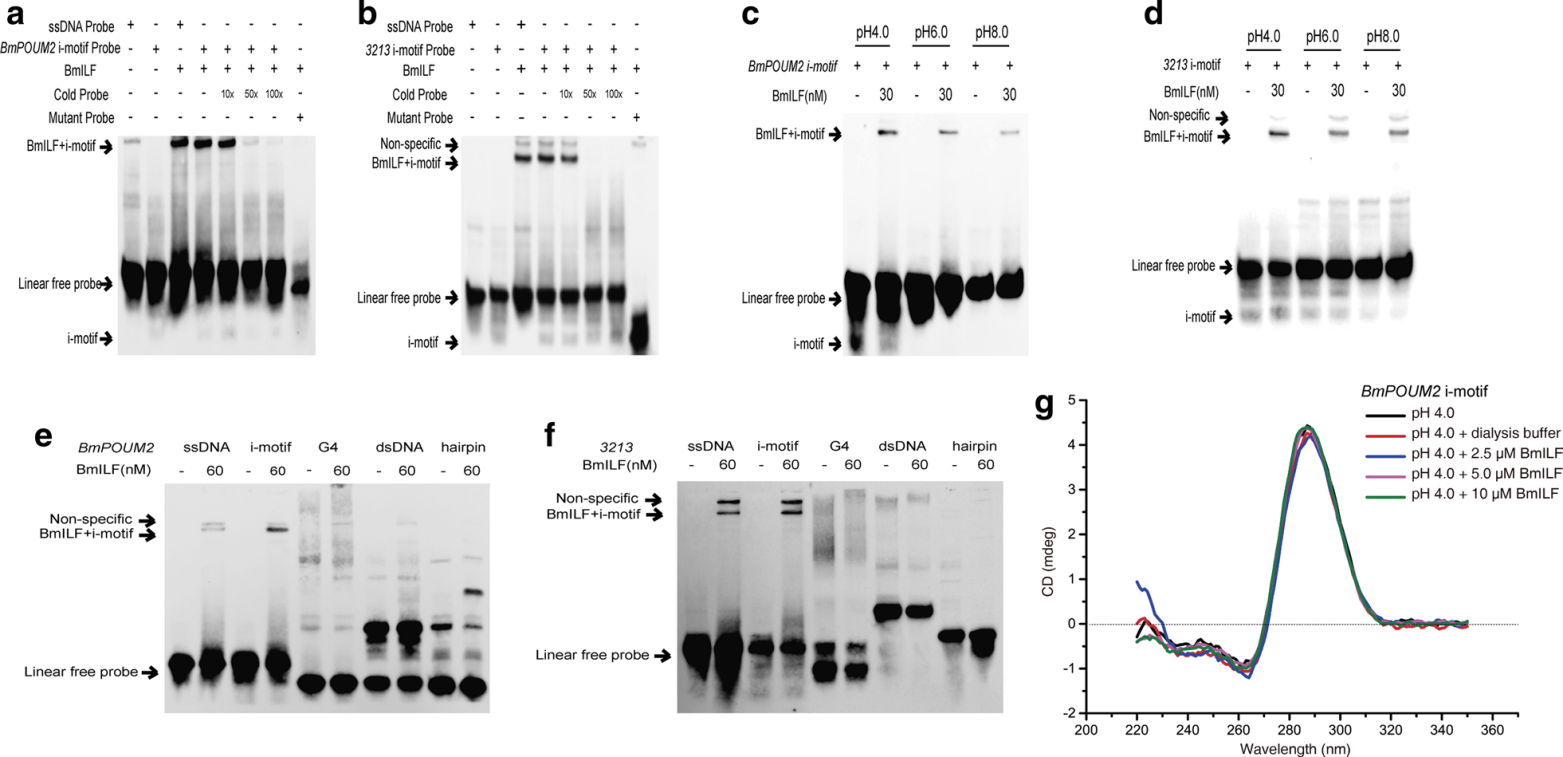
EMSA for the specific binding of BmILF to the i-motif structure. The i-motif probe was synthesized and refolded into an i-motif structure at pH 4.0, 6.0 and 8.0. The ssDNA is the unfolded sequence. The cold probe is the un-labeled i-motif probe. The sequence of the mutated probe is shown in Table 1. The linear free probe is the same DNA fragment that did not form an advanced structure during the annealing cooling process. EMSA for the binding of recombinant BmILF to the i-motif probe of *BmPOUM2***a** and *BGIBMGA003213***b** at pH 4.00 in the 1 × binding solution (20 μl, 2.5% glycerol, 0.05% NP-40, 5 mM MgCl_2_, 4 mM EDTA, recombinant BmILF protein and biotinylated end-labeled probe) at room temperature for 20 min. The running buffer contained 0.04 M Tris, 0.04 M H_3_BO_3_, 0.001 M EDTA-2Na and was filtered with 0.22-µm pore-size filter. EMSA for the binding of recombinant BmILF to the i-motif structure of *BmPOUM2* (**c**) and *BGIBMGA003213* (**d**) or the linear ssDNA probe at pH 4.0, 6.0 and 8.0. EMSA for the binding of recombinant BmILF to different DNA motifs on *BmPOUM2* (**e**) and *BGIBMGA003213* (**f**). The positions of the labeled i-motif-containing probe, labeled ssDNA probe, labeled bound i-motif and BmILF are shown by the arrows. **g** CD analysis of the complex of BmILF and i-motif at pH 4.0. The sequences of all the probes used in this figure are listed in Table 1

In our previous study [10], the binding of BmILF to the i-motif structure in the *BmPOUM2* promoter was demonstrated by EMSA and ChIP methods. In this study, the specific binding of BmILF to the i-motif structures of *BmPOUM2* and *3213* was confirmed by EMSA (Fig. 2). BmILF bound the i-motif structure of *BmPOUM2* and *3213,* and the binding could be suppressed by increasing cold probe concentration (Fig. 2a, b). The protein could not bind the mutant probes. With the increase in pH value, the specific binding gradually declined (Fig. 2c, d). These results suggest that the binding of BmILF to the i-motif structure of both the *BmPOUM2* and *3213* genes was affected by pH. BmILF had high affinity for the DNA i-motif, but it did not bind with hairpin sequence, dsDNA or G4 structure (Fig. 2e, f). It is noticed that a band binding to BmILF was also observed in the ssDNA samples (Fig. 2e, f). It is probably because the i-motif structure may be formed when the ssDNA probe is synthesized and it is hard to completely prevent the formation of i-motif structure in the presence of ILF protein. Another possibility is that the binding region of ssDNA likely constitutes the i-motif structure. In addition, some bands were found for the hairpin structure of a different sequence in the presence of BmILF (Fig. 2e), but we cannot explain it at this time. To demonstrate the existence of i-motif structure in the complex with BmILF, a CD analysis was performed (Fig. 2g). The results showed that incubation of BmILF with i-motif did not change the CD spectra of the i-motif structures, suggesting that the i-motif structures were in the complex with BmILF and the protein could not change the structure. These results indicate that BmILF is an i-motif structure-specific binding protein.

### Binding affinity of BmILF with i-motif structures

**Fig. 3:**
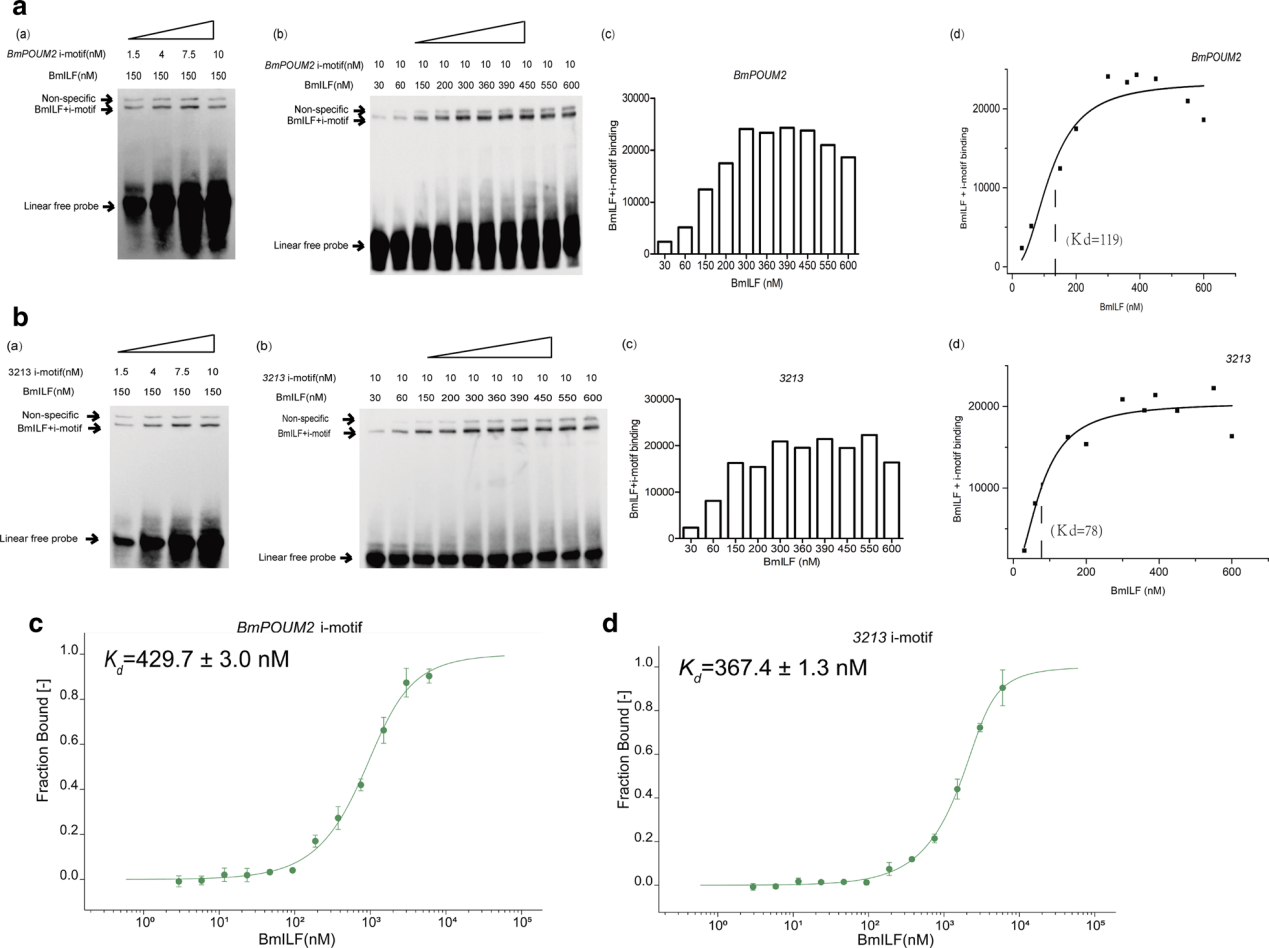
The binding affinity of BmILF to i-motif structures. EMSA for the binding of the recombinant BmILF to the i-motif probes of *BmPOUM2* (**A**) and *BGIBMGA003213* (**B**) at different concentrations of the probe (**Aa**, **Ba**) or protein (**Ab**, **Bb**). Quantitative measurement of the binding band of BmILF and i-motif of BmPOUM2 (**Ac**) and BGIBMGA003213 (**Bc**). Hill curves and the *K*_d_ values of the binding of BmILF and i-motif probes of *BmPOUM2* (**Ad**) and *BGIBMGA003213* (**Bd**). MST analysis for the binding affinity of the recombinant BmILF with the i-motif of *BmPOUM2* (**C**) and *BGIBMGA003213* (**D**) at different protein concentrations

With an increase in the probe or protein concentration, the specific binding was gradually strengthened (Fig. 3Aa, b, Ba, b). After numeralizing the intensity of the BmILF and i-motif complex bands (Fig. 3Ac, Bc), Hill curves of BmILF–i-motif binding were obtained and the *K*_d_ of the BmILF binding with the i-motif structure of *BmPOUM2* and *3213* was calculated to be 119 nM and 78 nM (Fig. 3Ad, Bd), respectively, which are close enough to the affinity (59 nM) for the binding between the iMab protein and the hTelo i-motif structure reported by Zeraati et al. [16]. To further confirm the binding affinity between BmILF and different i-motif structures, MST method, which is used to measure intermolecular interactions in solution [24], was conducted. As shown in the Fig. 3C, D, when the concentration of the i-motif probe was set at 50 nM, the *K*_d_ values of BmILF binding with the *BmPOUM2* and *3213* i-motif were 429.7 and 367.4 nM, respectively, 3.6 and 4.7 times higher than that by EMSA method, respectively. In other words, the binding affinities measured by EMSA were higher than those measured by the MST method.

### In vivo visualization of i-motif structure in testis

**Fig. 4:**
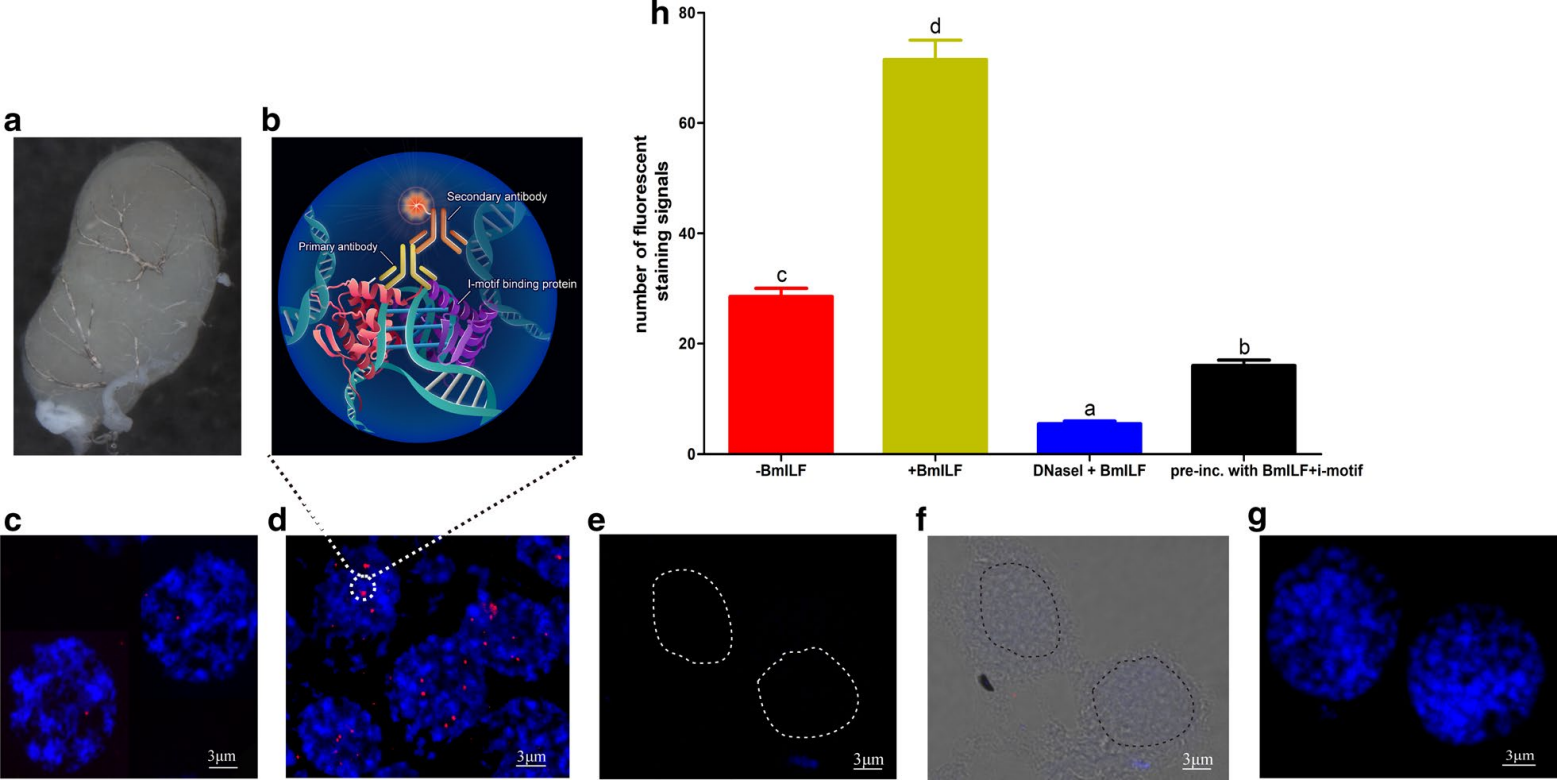
Immunofluorescence visualization of i-motif structure in the *B. mori* testis. **a** Testes were isolated from the 5th instar larvae and treated as described in “Materials and Methods” section. **b** Schematic diagram of i-motif immunofluorescence detection. The primary antibody was an anti-BmILF antibody at a 1:2000 dilution; the secondary antibody was Alexa Fluor™ 594-conjugated goat anti-rabbit IgG at a 1:400 dilution. The red fluorescence shows the binding of BmILF to the i-motif structure in the nuclei of testis cells. **c** BmILF antibody recognizes the endogenous BmILF-bound i-motif structure in the nucleus, without the addition of exogenous BmILF. **d** Increased i-motif staining after incubation with exogenous recombinant BmILF. **e** Immunofluorescence staining of the i-motif after treatment with DNase I. The dotted lines show the boundaries of the nuclei; **f** the white light and fluorescence merged image of **e**, showing the position of the nuclei. **g** Immunofluorescence staining of the i-motif after the pre-incubation of BmILF with pre-folded C-quadruplex oligonucleotides. Nuclei were counterstained with DAPI (blue). **h** Quantification of i-motif signals for 50 nuclei in five random observation regions on the slide. The data are represented as the mean ± SEM of three replicates

To examine whether i-motif structures can form in vivo, i-motif structures were examined in *B. mori* tissues. Testes were isolated from 5th instar larvae and fixed on a glass slide (Fig. 4a). The i-motif and BmILF complex was recognized by the specific BmILF antibody (Fig. 4b). The results showed that a few immunofluorescence signals were detected even without the addition of exogenous BmILF protein due to the presence of endogenous BmILF protein in the nuclei of testis cells (Fig. 4c). When exogenous BmILF was added, many more punctate fluorescence signals were seen (Fig. 4d). Treatment with DNase I, which digests DNA molecules, could abrogate the immunofluorescence signals (Fig. 4e, f), suggesting the natural presence of the i-motif and BmILF in the testis. The fluorescence signals disappeared when the BmILF protein was pre-incubated with an excess of pre-folded i-motif oligonucleotides prior to immunostaining (Fig. 4g), because the BmILF-i-motif oligo complex was washed off before the addition of the secondary antibodies. The statistical counts of the immunofluorescence signals under these four conditions are shown in Fig. 4h. These results indicated that BmILF can recognize and bind i-motif structures in vivo.

### Visualization of i-motif structures in chromosomes during the testis cell cycle

**Fig. 5:**
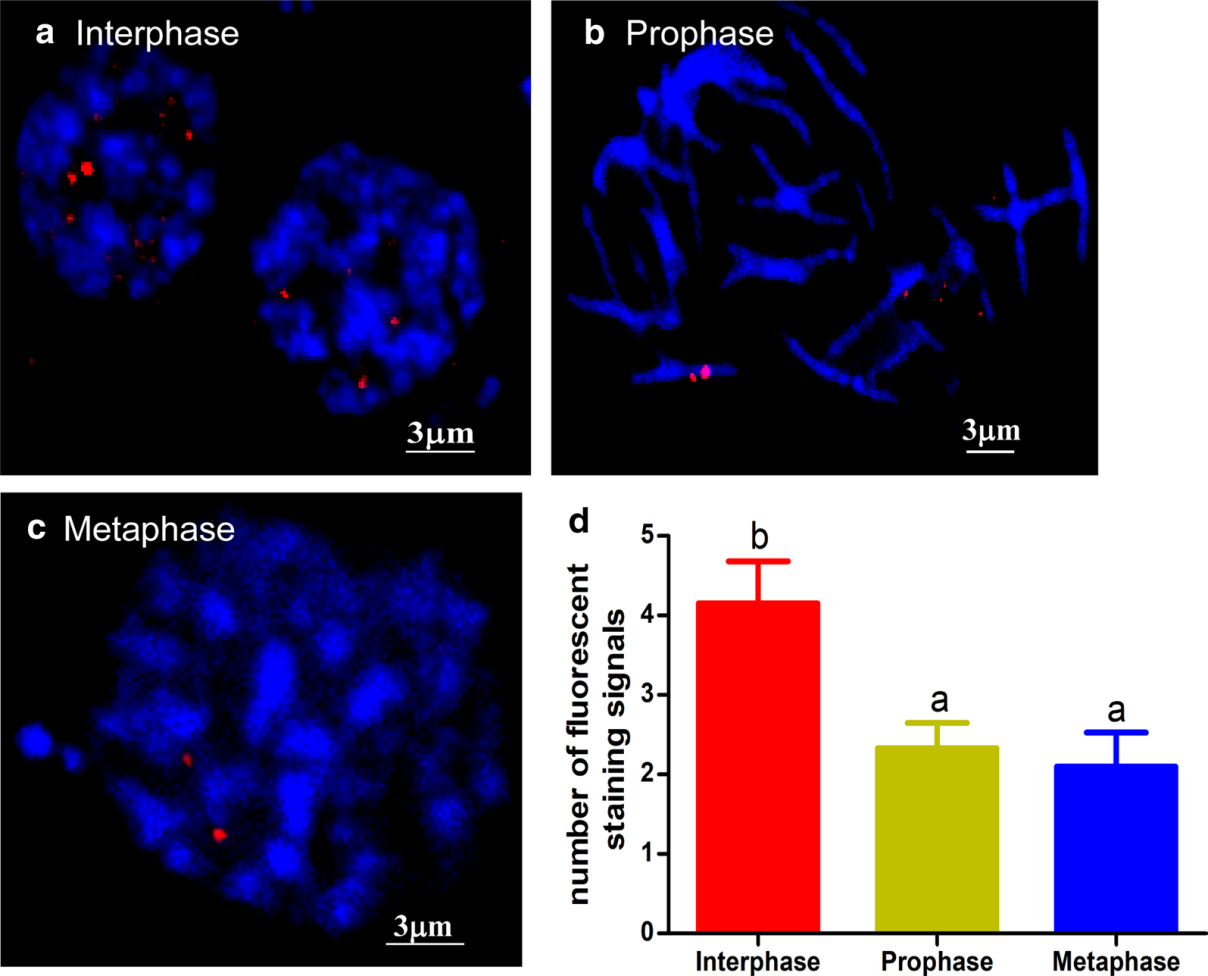
Immunofluorescence detection of i-motif structures in *B. mori* chromosomes during the cell cycle in testis. Testes were isolated from the 4th instar larvae and treated as described in “Materials and Methods” section. **a**–**c** Staining of the i-motif (red) was observed in the interphase, prophase and metaphase of the cell cycle. **d** Quantification of the average number of i-motif staining signals for nuclei of testis cells. The data are represented as the mean ± SEM of three replicates. Chromosomes were counterstained with DAPI (blue)

The presence of the i-motif was also observed in chromosomes at different stages of the testis cell cycle (Fig. 5). There were twice as many i-motif structures detected in interphase (Fig. 5a) than in prophase (Fig. 5b) and metaphase (Fig. 5c). The statistical analysis of the fluorescence signals is shown in Fig. 5d. These observations indicate that there are more i-motif structures formed when the chromatin is in a loose state, implying that these detected i-motif structures may be involved in the regulation of gene expression in the cells.

### Effects of porphyrin compounds on the formation of i-motif structures in testis

**Fig. 6:**
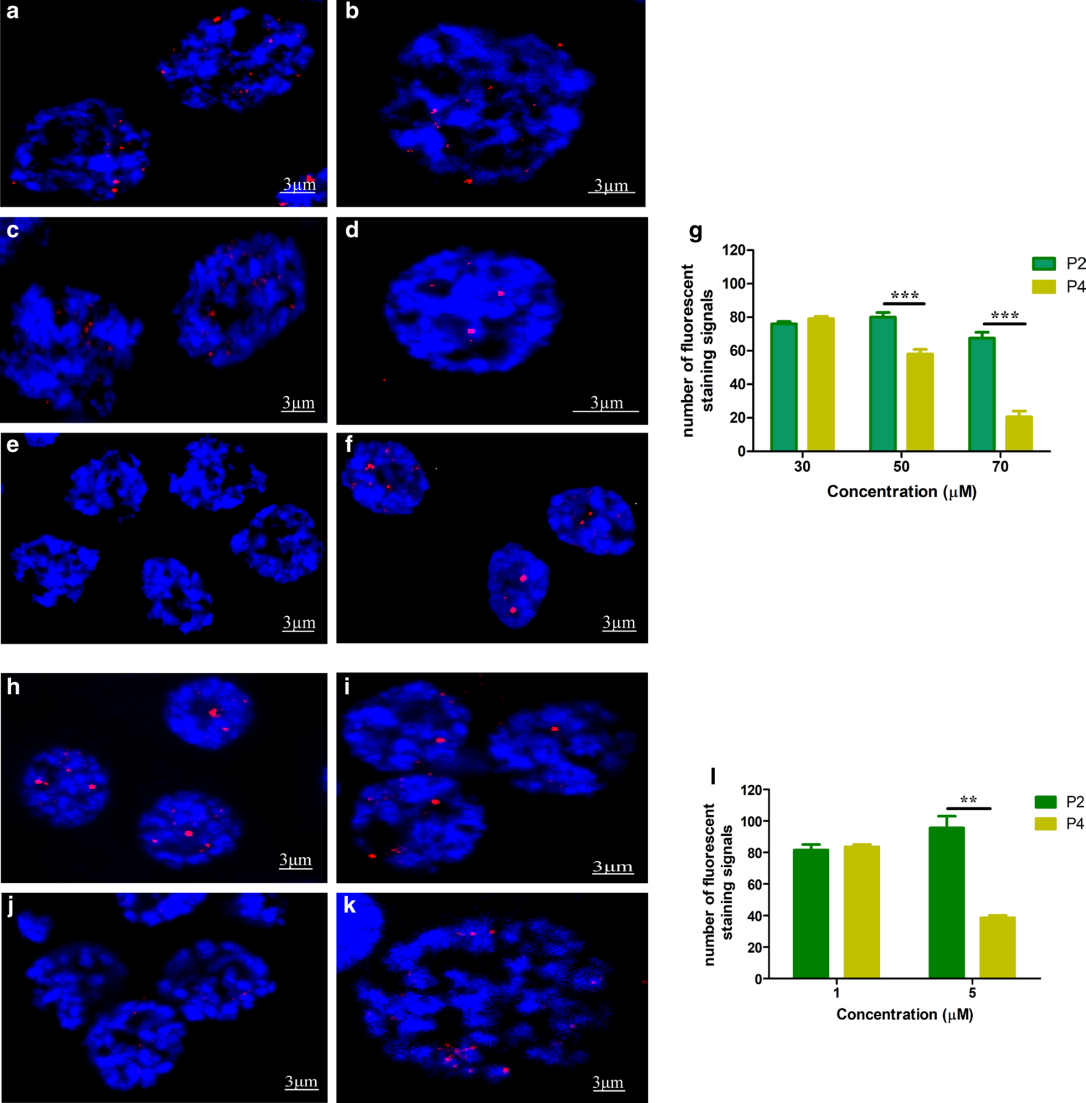
Effects of porphyrin compounds on i-motif formation in *B. mori* testis cells. Testes were isolated from 5th instar larvae and treated as described in “Materials and Methods” section. The porphyrin compound TMPyP4 (**a**, **c**, **e**, **h** and **j**) or TMPyP2 (**b**, **d**, **f**, **i** and **k**) at 1 (**h**, **i**), 5 (**j**, **k**), 30 (**a**, **b**), 50 (**c**, **d**) or 70 µM (**e**, **f**) was added to the testis culture medium before immunofluorescence analysis. The red fluorescence signals show the binding of BmILF to i-motif structures in the nuclei. The nuclei were counterstained with DAPI (blue). **g**, **l** Quantification of i-motif staining signals for 50 nuclei in five randomly selected observation regions on the slide. The data are represented as the mean ± SEM of three replicates

The porphyrin compound TMPyP4 has been reported to affect the formation of i-motifs [25, 26], while TMPyP2 has no effect on the structure [9, 27]. To test whether TMPyP4 can reduce the formation of i-motif structures in testis cells, the cells were treated with TMPyP4 or TMPyP2 prior to incubation with BmILF and its antibody. At 30 μM (Fig. 6a), TMPyP4 had no significant influence on the i-motif structures. However, when the TMPyP4 concentration was increased to 50 (Fig. 6c) or 70 μM (Fig. 6e), the number of i-motif structures was significantly reduced, whereas TMPyP2 treatment did not cause a significant change in the fluorescence signals (Fig. 6b, d and f). To further confirm the effect of the concentration of these compounds, lower concentrations (1 and 5 µM) were tested. There was no difference in the fluorescence signal for treatment with TMPyP4 or TMPyP2 at 1 µM (Fig. 6h, i). At 5 µM, the fluorescence with TMPyP4 treatment was significantly lower than that with TMPyP2 treatment (Fig. 6j, k). The statistical counts of the immunofluorescence signals are shown in Fig. 6g, l. These results suggest that TMPyP4 may destroy the i-motif structures that BmILF binds to.

### Effects of pH on the formation of i-motif structures in testis

**Fig. 7:**
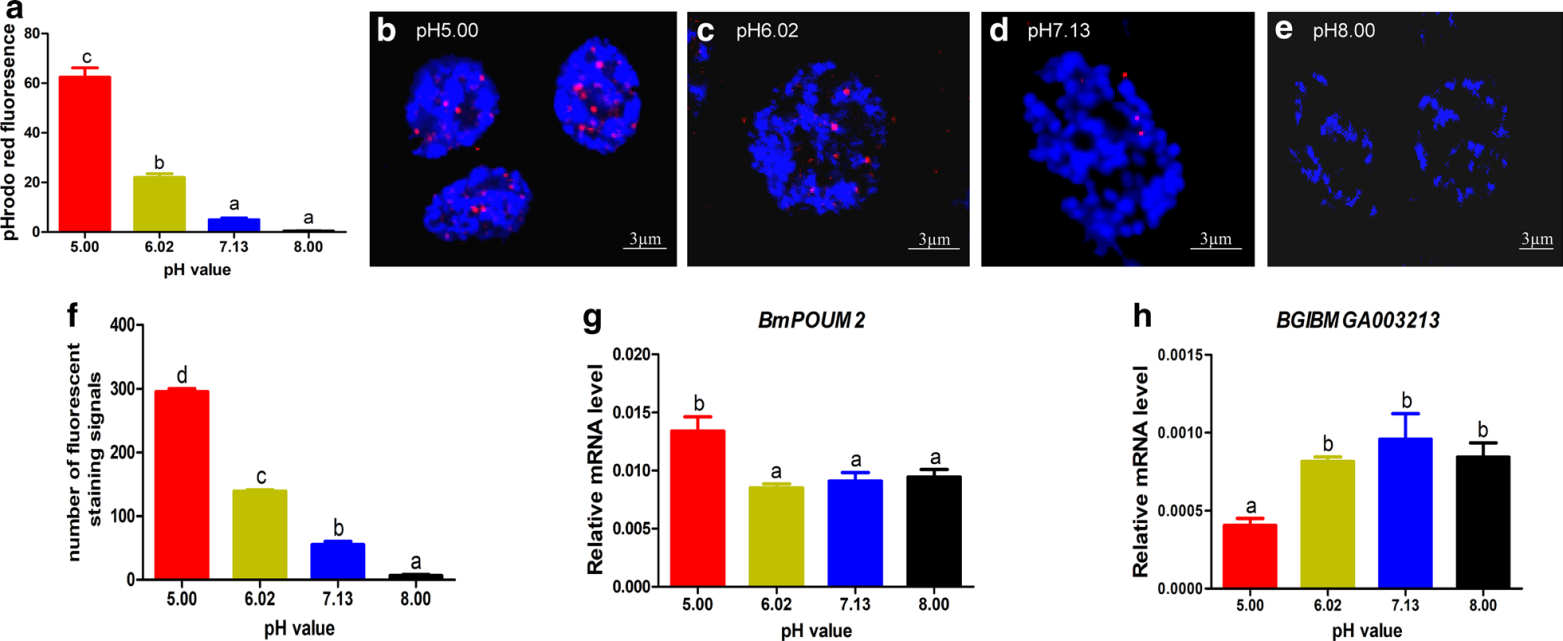
Effects of different pH values on i-motif formation in the *B. mori* testis. Testes were isolated from 5th instar larvae and treated as described in “Materials and Methods” section. **a** Analysis of the intracellular pHrodo Red fluorescence intensity in testis cells cultured in media with different pH values. A higher fluorescence intensity indicates a lower pH value. **b**–**e** The pH values of the testis cell culture medium were adjusted with HCl and NaOH to 5.00, 6.02, 7.13 and 8.00 before immunofluorescence detection. The red fluorescence signals show the binding of BmILF to i-motif structure. Nuclei were counterstained with DAPI (blue). **f** Quantification of i-motif staining signals for 50 nuclei in five randomly selected observation regions on the slide. The data are represented as the mean ± SEM of three replicates. **g** qRT-PCR analysis of the pH effects on the expression of *BmPOUM2*. **h** qRT-PCR analysis of the pH effects on the expression of *BGIBMGA003213*

To test whether pH changes can alter the i-motif numbers in the nuclei, testes were cultured in vitro in insect medium with different pH values before the examination of i-motif structures by immunostaining. The change in the intracellular pH of the cells cultured in medium with different pH values was examined using pHrodo Red as an intracellular pH indicator (Fig. 7a). The results revealed that the fluorescence signals decreased with an increase in the pH of the culture medium (Fig. 7b–e). The statistical counts of the immunofluorescence signals in the cells under different pH conditions are shown in Fig. 7f.

To test whether the change in the i-motif structure affects gene expression, the testes were isolated from the 5th instar larvae and cultured in vitro under different pH conditions. Then, the expression of the target genes, *BmPOUM2* and *3213*, which contain the i-motif structure, was analyzed. The results showed that the expression of *BmPOUM2* decreased with increasing pH (Fig. 7g), in contrast, the expression of *3213* increased (Fig. 7h). These results suggest that the formation of i-motif structures might be positively or negatively involved in the transcription regulation of target genes.

## Discussion

DNA is usually present in living cells as a double-helix structure [1]. However, it has been found that non-double-helix secondary structures such as G4 and i-motif structures are also present in DNA molecules in some cases [2, 28, 29]. The in vivo presence of G4 structures has been detected in several studies using immunofluorescence staining methods [10, 13]. Although it has been long demonstrated that C-rich sequences can form i-motif structures under in vitro conditions [7, 8], in vivo evidence of the presence of i-motif structures in living cells was lacking until a recent report, in which the i-motif structure was detected for the first time in vivo in a living human cell line [16]. Zeraati et al. used an antibody fragment (iMab) that recognizes i-motif structures with high selectivity and affinity to detect the presence of i-motifs in the nuclei of human cells. They found that the in vivo formation of the i-motif is cell cycle and pH dependent [16]. In the present study, the existence of i-motif structures in the testis cells of invertebrate silkworms was demonstrated using an antibody generated against BmILF that specifically binds to i-motif structures in the promoter regions of *BmPOUM2* and *3213* genes (Figs. 2–4). Thus, it is clear that i-motif structure is present in vivo in cells of not only vertebrates but also invertebrates, suggesting the importance of this motif as a regulatory mechanism of gene transcription.

In this study, two methods, EMSA and MST, were used to measure the binding affinities of the BmILF and i-motif structures. Although these two methods gave different *K*_d_ valves (119 vs 429.7 nM for *BmPOUM2* i-motif and 78 vs 367.4 nM for *3213* i-motif), approximately three- to fourfold higher in MST than those in EMSA, they are at the same nM order of magnitude and reasonably similar to that (59 nM) found in the binding between iMab protein and the hTelo i-motif structure in human cells by Biolayer Interferometry method [16], indicating that the BmILF binding with i-motif structure is highly specific and affinitive, although not as high as that between iMab protein and the hTelo i-motif in human. The different affinities of BmILF for the *BmPOUM2* i-motif and *3213* i-motif are probably due to different length of the loops of the two structures, which will be clarified in future.

Many studies have shown that pH has an important effect on the formation of the i-motif [7, 8, 30]. Acidic conditions favor i-motif formation, whereas alkaline conditions favor its deformation, because the formation of the C–C^+^ bonds that make up the i-motif conformation requires an acidic environment [29, 31, 32]. Thus, any changes in the cellular pH may affect the formation and deformation of the i-motif. In this study, the effects of pH on the formation of i-motif structures in the *BmPOUM2* and *3213* genes (Fig. 1) and the binding of these i-motifs to BmILF in vitro (Fig. 2) and in vivo (Fig. 7) were analyzed, and the results revealed that decreasing pH, the structure was formed and bound by BmILF, thus confirming the finding in human cells [16]. In addition, the pH in cells may depend on the cellular status, which is influenced by cellular physiological and environmental factors [33–35]. Therefore, the i-motif-mediated regulation of gene expression can be considered as an epigenetic mechanism, which depends on the DNA secondary structure as well as physiological and environmental factors, but not on the genetic sequence and codes.

If the i-motif structure is involved in the regulation of gene transcription, it should form in the active phases of cellular and DNA activities. Zeraati’s studies [16] indicated that the most i-motif structures appear during the interphase of mitosis in human cell line. Our results confirmed Zeraati’s results. This consistency implies that the i-motif may be associated with the regulation of gene transcription. Additional evidence for the involvement of the i-motif in the regulation of gene expression comes from the experiments on target gene expression, which showed that when the i-motif structure was formed at lower pH values, it regulates the expression of the target genes *BmPOUM2* (positively) and *3213* (negatively), which contain the i-motif structure in their promoters (Fig. 7). These results strongly suggest that i-motif structures participate in the regulation of gene expression in the nuclei of cells during interphase.

It must be noted that in this study, BmILF and its antibody were used to detect the in vivo presence of i-motif structures in the testis (Fig. 4). Although the specificity of BmILF binding with the i-motif structures in *BmPOUM2* and *3213* was confirmed by EMSA (Fig. 2), it is not known whether BmILF can bind other types of i-motif structures in other genes. In a search of the *B. mori* genome, only one copy of the *BmPOUM2* and *3213* genes was found. However, immunostaining showed multiple punctate fluorescence signals in the nuclei or chromosomes of the testis, suggesting that BmILF may be able to recognize other types of i-motif structures in other genes in the *B. mori* genome, in which there are 6278 potential predicted G4/i-motif sequences [10].

We also note that sometimes the BmILF protein bound with the i-motif-containing ssDNA (Fig. 2e, f) or at high pH condition (Fig. 2c, d), at which the i-motif structure is not supposed to form. Thus, the possibility of the BmILF binding with the i-motif containing linearized DNA cannot be completely ruled out. Kang et al. [36] found that the BCL2 promoter contains an i-motif structure, which is highly dynamic in nature and can form a flexible hairpin [36]. Transcriptional factor hnRNP LL binds with the two loops of the i-motif structure, unfolding the structure and activating the BCL2 expression. Lannes et al. [37] further demonstrated that the RRM12 regions of hnRNP LL recognize a single-stranded CTCCC element present in the two loops of the i-motif [37]. These loop regions are single-stranded DNA that are parts of the i-motif structure. The nature and characters of the BmILF binding with the i-motif structure as well as the DNA sequence still need to be further investigated.

## Conclusions

This study for the first time confirmed the in vivo existence of the i-motif structure, which depends on the pH and cell cycle, in the testis cells of invertebrate silkworms and demonstrated that the i-motif structure is involved in transcriptional regulation of the target genes. This work provides new insights into epigenetic regulatory mechanisms of gene expression by DNA secondary structures in the silkworm, a model lepidopteran insect.

## Materials and methods

### Isolation of *B. mori* testes

*B. mori* testes were carefully isolated from 4th and 5th instar larvae under a microscope. The fat body and trachea were carefully removed. The testes were washed three times before in vitro culture in 96-well plates.

### In vitro culture of *B. mori* testes and intracellular pH detection

The isolated *B. mori* testis were cultured in Grace’s insect medium with 10% serum. The original pH value of the medium was 6.02, and the pH was adjusted using HCl and NaOH to 5.00, 7.13 or 8.00 depending on the assay. The tissues were cultured in the wells of 96-well plates at 28 ℃ for 36 h before analysis. For the TMPyP4 (5,10,15,20-tetrakis-(*N*-methyl-4-pyridyl)porphyrin) and TMPyP2 (5,10,15,20-tetrakis-(*N*-methyl-2-pyridyl)porphyrin) treatment, the medium was adjusted to pH 6.02, and TMPyP4 or TMPyP2 was added to a final concentration of 1, 5, 30, 50 and 70 μm. The samples were analyzed after 36 h of culture.

The intracellular pH of the testis cells after tissue culture was determined using the pHrodo Red AM Intracellular pH Indicator (Thermo Scientific, Eugene, USA). The pHrodo^®^ Red dye is modified with AM ester groups, which causes uncharged molecules to penetrate the cell membrane. Upon entry into the cell, the lipophilic blocking group is cleaved by a non-specific esterase, resulting in a compound that remains in the intracellular space. The fluorescence intensity of the probe is then an indicator of the intracellular pH. The cultured testes were washed 3 times with PBS and then stained in pHrodo Red dye for 30 min. The dye was removed, and the tissues were washed 3 times with PBS. Digital images were acquired using an FV3000 camera connected to an IX83 microscope (Olympus) and analyzed with cellSens software (Olympus).

### RNA isolation and qRT-PCR

Total RNA was isolated from the testes after culture and treatment using the Eastep^®^ Super Total RNA Extraction Kit (Promega, Beijing, China). Quantitative real-time PCR (qRT-PCR) was conducted using a GoScript™ Reverse Transcription Mix, Random Primers Kit (Promega, Beijing, China). qRT-PCR was performed with the following reagents under the following conditions: SYBR Premix Ex Taq (× 2): 10 µl in a 20 µl reaction volume; the primer concentrations: 0.8 µl (10 µM); immunoprecipitated DNA samples: 2 µl. The mixtures were incubated at 95 ℃ for 2 min followed by 40 cycles at 95 ℃ for 15 s and 60 ℃ for 40 s. A QuantStudio 6 Flex fluorescence quantitative PCR system was used.

### Circular dichroism (CD) analysis

CD analyses were performed with a J-815 CD spectrometer (Jasco International, USA). All spectra were collected over a wavelength range of 220–350 nm, with a 1 nm step width and 1 s response time. The CD spectra are representations of three averaged scans of each sample taken at room temperature and are baseline corrected for signal contributions due to the buffer. DNA oligonucleotide sequences (Table 1) were diluted to 5 μM in 50 mM Tris–acetate buffer (Tris–TAE buffer) at pH 5.00, 6.02, 7.13 and 8.00. To fold the ssDNA into the i-motif conformation, the DNA sequences were heated at 95 ℃ for 10 min and then slowly cooled to room temperature over a 4-h period before CD scanning.

**Table 1:** i-motif, G4, hairpin and mutant sequences used in this study

Gene ID	Wild-type and mutant sequences of the i-motif, G4 and hairpin^a^	Used for
BmPOUM2-WT	TTGTTGCCCCGCCCCTCGGCCCCCTCGCGCCCCGCACTGG	BmPOUM2-WT in Fig. 1a; ssDNA, i-motif and cold probes in Fig. 2a; i-motif in Fig. 2c, Fig. 2e, Fig. 2g and Fig. 3A(a,b) and Fig. 3C; ssDNA in Fig. 2e.
BmPOUM2-Mut	TTGTTGTACTGCTACTCGGATCACTCGAGCATCGTACTGG	Mutant probes in Figs. 1b, 2a
BGIBMGA003213-WT	CCCCGCGCCCCCCGCGTCCCCCGCACCCCC	BGIBMGA003213-WT in Fig. 1c; ssDNA, i-motif and cold probes in Fig. 2b; i-motif in Figs. 2d,f, 3B(a,b), D; ssDNA in Fig. 2f.
BGIBMGA003213-Mut	CATTGCGATATCAGCGTTATCAGTATCAAT	Mutant probes in Figs. 1d, 2b
BmPOUM2-G4	CCAGTGCGGGGCGCGAGGGGGCCGAGGGGCGGGGCAACAA	BmPOUM2 G4 in Fig. 2e
BGIBMGA003213-G4	GGGGGTGCGGGGGACGCGGGGGGCGCGGGG	3213 G4 in Fig. 2f
Hairpin	CAGTGGCGGCCATCCGTTTAATATTACCGGATGGCCGCGCGAT	Hairpin in Fig. 2e, f

Hairpin was synthesized based on the tomato golden mosaic virus sequence. The i-motif, G4 and mutant sequences were synthesized based on the sequences of *BmPOUM2* and *BGIBMGA003213* genes

^a^The underlined based are mutated

### Preparation of recombinant BmILF protein and its antibody

The preparation method for recombinant BmILF protein was described in our previous study [11]. The BmILF ORF was inserted into the pET-28a expression vector, and the recombinant protein was expressed in *E. coli* cells (BL21). The protein was purified with Ni affinity chromatography using the His-Bind^®^ Kit according to the manufacturer’s protocol. The purified antigen protein was used to immunize rabbits by abdominal injection. Three injections were performed, each with 0.5–1 mg of protein, at 10–14 day intervals. After the three immunizations, antiserum was collected and the titer and specificity of the antibody were examined by Western blotting against testis tissue proteins, confirming the high specificity of the BmILF antibody.

### Electrophoretic mobility shift assay (EMSA) for protein–DNA binding

EMSA was conducted using a Light Shift Chemiluminescent EMSA Kit (Thermo Scientific, Waltham, USA). A hairpin sequence was constructed based on the tomato golden mosaic virus sequence [16]. The i-motif, single-stranded DNA and double-stranded DNA containing the G4 sequence were constructed based on the *BmPOUM2* or *BGIBMGA003213* sequences. The oligonucleotides were labeled with biotin at the 5 end and heated at 95 ℃ for 10 min at pH 4.0, 6.0 and 8.0 in 50 mM Tris–TAE buffer and slowly cooled to room temperature over 4 h. The oligonucleotides labeled with biotin at the 5′ end were synthesized by Tsingke (Guangzhou, China).

Binding reactions were performed according to the instructions of the EMSA kit. In brief, reactions were conducted in a 20 μl volume of binding buffer (2.5% glycerol, 0.05% NP-40, 5 mM MgCl_2_, 4 mM EDTA, recombinant BmILF protein and biotinylated end-labeled probe) at room temperature for 20 min. The samples were then separated on a 12% polyacrylamide gel on the ice at 100 V for 3 h. The running buffer for EMSA contained 0.04 M Tris, 0.04 M H_3_BO_3_, 0.001 M EDTA-2Na and was filtered with 0.22-µm pore-size filter. After electrophoresis, the gel was blotted onto a positively charged nylon membrane (Amersham Biosciences, Waltham, USA). Membranes were then developed using the EMSA kit according to the manufacturer’s protocol. The intensity of the binding bands in EMSA was scanned using ImageJ software and numeralized. Hill curves of the values related to the BmILF protein concentrations were prepared using OriginPro software to obtain *K*_d_ values. The oligonucleotide probes used in this study are shown in Table 1.

### Microscale thermophoresis (MST)

The DNA oligonucleotides (Table 1) were labeled with Cy5 and heated at 95 ℃ for 10 min in 50 mM Tris buffer at pH 4.0 and slowly cooled to room temperature overnight. Purified BmILF at different concentrations of 2.93–6000 nM and 50-nM labeled i-motif structures were incubated for 20 min at room temperature in the Nanotemper buffer (NanoTemper Technologies GmbH, Munich, Germany) containing 100 mM Tris (pH 6.0). MST analysis was performed using a standard capillary from NanoTemper and on a Monolith NT.115 column with 60% LED and 40% MST power at 25 ℃. All experiments were repeated three times for each measurement. Data analysis was performed using NanoTemper analysis software.

### Immunofluorescence analysis of i-motif structures in cells

Testes were carefully isolated from the 4th and 5th instar larvae of *B. mori,* and fat body and trachea were removed from the testes. The testes were washed with a hypotonic solution of 0.075 mol/L KCl at 37 ℃ three times, for 45 min each. The testes were then ground and fixed in Carnoy’s fixative for 2 h [21, 22]. After blocking with 1% goat serum, the experimental group was incubated with purified BmILF protein (8 mg/ml) in 1% goat serum and the control group was incubated in 1% goat serum. After a 3-h incubation, the samples were washed with 0.4% phosphate-buffered solution containing Triton™ X-100 (PBST) at least three times, for 10 min each. Anti-BmILF antibody at a dilution of 1:2000 was added and incubated at 4 ℃ overnight. The samples were washed with 0.4% PBST three times, 15 min each, and the secondary antibody (Alexa 594-conjugated antibody) was then added at a dilution of 1:400 and incubated for 1 h before 3 PBST washes of 10 min each. The samples were mounted on glass using ProLong gold antifade reagent and stained with DAPI [23]. Digital images were recorded using an FV3000 camera connected to an IX83 microscope (Olympus) and analyzed with cellSens software (Olympus).

For the pre-incubation of the BmILF protein with an excess of pre-folded i-motif oligonucleotides, the wild-type and mutant oligonucleotides were heated at 95 ℃ for 10 min at pH 4.0 in 50 mM Tris–TAE buffer and slowly cooled to room temperature over 4 h. When the chromosome was blocked, 8 mg/ml BmILF was incubated with an excess of pre-folded i-motif oligonucleotides at room temperature for 30 min and was then added to the experimental group as follow-up experiment.

For the DNase I treatment, after the testes were fixed on a glass slide, the experimental group was treated with DNase I at room temperature for 1 h and then washed with 0.4% PBST three times for 10 min each.

For the statistical counts of the immunofluorescence signals, five observation regions were randomly selected on each slide and the number of fluorescent-stained nuclei in a total of 50 nuclei was counted under a microscope with a × 100 objective. Each sample was analyzed three times, and the counts are represented as averages.

## Data Availability

Data sharing not applicable to this article as no datasets were generated or analyzed during the current study. The amino acid sequence of the transcription factor BmILF is deposited in GenBank (GenBank accession no.: KY082711).

## References

[1] Watson JD, Crick FH (1953). Molecular structure of nucleic acids; a structure for deoxyribose nucleic acid. Nature.

[2] Huppert JL, Balasubramanian S (2007). G-quadruplexes in promoters throughout the human genome. Nucleic Acids Res.

[3] Cogoi S, Xodo LE (2006). G-quadruplex formation within the promoter of the KRAS proto-oncogene and its effect on transcription. Nucleic Acids Res.

[4] Leroy JL, Gueron M, Mergny JL, Helene C (1994). Intramolecular folding of a fragment of the cytosine-rich strand of telomeric DNA into an i-motif. Nucleic Acids Res.

[5] Snoussi K, Nonin-Lecomte S, Leroy JL (2001). The RNA i-motif. J Mol Biol.

[6] Dettler JM, Buscaglia R, Cui J, Cashman D, Blynn M, Lewis EA (2010). Biophysical characterization of an ensemble of intramolecular i-motifs formed by the human c-MYC NHE III1 P1 promoter mutant sequence. Biophys J.

[7] Zhou J, Wei CY, Jia GQ, Wang XL, Feng ZC, Li C (2010). Formation of i-motif structure at neutral and slightly alkaline pH. Mol BioSyst.

[8] Wright EP, Huppert JL, Waller ZAE (2017). Identification of multiple genomic DNA sequences which form i-motif structures at neutral pH. Nucleic Acids Res.

[9] Banerjee K, Wang M, Cai E, Fujiwara N, Baker H, Cave JW (2014). Regulation of tyrosine hydroxylase transcription by hnRNP K and DNA secondary structure. Nat Commun..

[10] Niu K, Zhang X, Deng H, Wu F, Ren Y, Xiang H (2018). BmILF and i-motif structure are involved in transcriptional regulation of BmPOUM2 in *Bombyx mori*. Nucleic Acids Res.

[11] Abou Assi H, Garavis M, Gonzalez C, Damha MJ (2018). i-Motif DNA: structural features and significance to cell biology. Nucleic Acids Res.

[12] Dembska A (2016). The analytical and biomedical potential of cytosine-rich oligonucleotides: a review. Anal Chim Acta.

[13] Murat P, Singh Y, Defrancq E (2011). Methods for investigating G-quadruplex DNA/ligand interactions. Chem Soc Rev.

[14] Guo K, Gokhale V, Hurley LH, Sun D (2008). Intramolecularly folded G-quadruplex and i-motif structures in the proximal promoter of the vascular endothelial growth factor gene. Nucleic Acids Res.

[15] Biffi G, Tannahill D, McCafferty J, Balasubramanian S (2013). Quantitative visualization of DNA G-quadruplex structures in human cells. Nat Chem..

[16] Zeraati M, Langley DB, Schofield P, Moye AL, Rouet R, Hughes WE (2018). I-motif DNA structures are formed in the nuclei of human cells. Nat Chem..

[17] Gehring K, Leroy JL, Gueron M (1993). A tetrameric DNA structure with protonated cytosine cytosine base pairs. Nature.

[18] Kumar N, Patowary A, Sivasubbu S, Petersen M, Maiti S (2008). Silencing c-MYC expression by targeting quadruplex in P1 promoter using locked nucleic acid trap. Biochemistry.

[19] Kendrick S, Kang HJ, Alam MP, Madathil MM, Agrawal P, Gokhale V (2014). The dynamic character of the BCL2 promoter i-motif provides a mechanism for modulation of gene expression by compounds that bind selectively to the alternative DNA hairpin structure. J Am Chem Soc.

[20] Dzatko S, Krafcikova M, Hansel-Hertsch R, Fessl T, Fiala R, Loja T (2018). Evaluation of the stability of DNA i-motifs in the nuclei of living mammalian cells. Angew Chem Int Ed Engl.

[21] Pan MH, Xiao SQ, Chen M, Hong XJ, Lu C (2007). Establishment and characterization of two embryonic cell lines of *Bombyx mori*. In Vitro Cell Dev Biol Anim..

[22] Gaffarzadeh-Namazi L, Asghari-Zakaria R, Babaeian N, Kazemi-Tabar K (2007). Comparative study of chromosome morphology and C-banding patterns in several genotypes of Lens culinaris. Pak J Biol Sci.

[23] Johansen KM, Cai W, Deng H, Bao X, Zhang W, Girton J (2009). Polytene chromosome squash methods for studying transcription and epigenetic chromatin modification in Drosophila using antibodies. Methods.

[24] Wienken CJ, Baaske P, Rothbauer U, Braun D, Duhr S (2010). Protein-binding assays in biological liquids using microscale thermophoresis. Nature Commun..

[25] Fedoroff OY, Rangan A, Chemeris VV, Hurley LH (2000). Cationic porphyrins promote the formation of i-motif DNA and bind peripherally by a nonintercalative mechanism. Biochemistry.

[26] Khan N, Avino A, Tauler R, Gonzalez C, Eritja R, Gargallo R (2007). Solution equilibria of the i-motif-forming region upstream of the B-cell lymphoma-2 P1 promoter. Biochimie.

[27] Grand CL, Han H, Munoz RM, Weitman S, Von Hoff DD, Hurley LH (2002). The cationic porphyrin TMPyP4 down-regulates c-MYC and human telomerase reverse transcriptase expression and inhibits tumor growth in vivo. Mol Cancer Ther.

[28] Parkinson GN, Lee MP, Neidle S (2002). Crystal structure of parallel quadruplexes from human telomeric DNA. Nature.

[29] Sen D, Gilbert W (1988). Formation of parallel four-stranded complexes by guanine-rich motifs in DNA and its implications for meiosis. Nature.

[30] Day HA, Huguin C, Waller ZA (2013). Silver cations fold i-motif at neutral pH. Chem Commun (Camb).

[31] Fujii T, Sugimoto N (2015). Loop nucleotides impact the stability of intrastrand i-motif structures at neutral pH. Phys Chem Chem Phys.

[32] Jin KS, Shin SR, Ahn B, Rho Y, Kim SJ, Ree M (2009). pH-dependent structures of an i-motif DNA in solution. J Phys Chem B..

[33] Huang Z, Huang Y (2005). The change of intracellular pH is involved in the cisplatin-resistance of human lung adenocarcinoma A549/DDP cells. Cancer Invest.

[34] Vogel M, Gunther A, Rossberg A, Li B, Bernhard G, Raff J (2010). Biosorption of U(VI) by the green algae *Chlorella vulgaris* in dependence of pH value and cell activity. Sci Total Environ.

[35] Webb BA, Chimenti M, Jacobson MP, Barber DL (2011). Dysregulated pH: a perfect storm for cancer progression. Nat Rev Cancer.

[36] Kang HJ, Kendrick S, Hecht SM, Hurley LH (2014). The transcriptional complex between the BCL2 i-motif and hnRNP LL is a molecular switch for control of gene expression that can be modulated by small molecules. J Am Chem Soc.

[37] Lannes L, Young P, Richter C, Morgne N, Schwalbe H (2017). Interaction of the N-terminal tandem domains of hnRNP LL with the BCL2 promoter i-motif DNA sequence. Chem Bio Chem.

